# Non-alcoholic Wernicke Encephalopathy During Orthopedic Rehabilitation in a Patient Following Total Gastrectomy: A Case Report

**DOI:** 10.7759/cureus.93023

**Published:** 2025-09-23

**Authors:** Shunsuke Ota, Yoshihisa Fujinami, Keiji Sato, Manabu Kirita

**Affiliations:** 1 Department of Emergency Medicine, Kakogawa Central City Hospital, Kakogawa, JPN

**Keywords:** aged, gastrectomy, non-alcoholic wernicke encephalopathy, thiamine deficiency, wernicke encephalopathy

## Abstract

An 82-year-old woman, who had undergone total gastrectomy for gastric cancer 22 years earlier, fell at home three months prior and underwent surgical repair for a femoral neck fracture. She had been receiving rehabilitation at a convalescent hospital. One week before presentation to our hospital, she experienced another fall, after which she developed decreased oral intake, generalized weakness, and altered mental status. Due to these worsening symptoms, she was transferred from the rehabilitation hospital to our facility.

After admission, brain magnetic resonance imaging was performed, which revealed high-signal intensities around the midbrain aqueduct and bilateral medial thalami on FLAIR imaging, leading to a diagnosis of Wernicke encephalopathy (WE). Her serum thiamine level was markedly low at 1.2 μg/dL. Although she had no history of alcohol use, WE was strongly suspected. Her mental status improved with intravenous thiamine administration and infusion therapy for dehydration. Two weeks later, she was transferred back to a rehabilitation facility for continued care.

In this case, chronic thiamine malabsorption due to prior total gastrectomy was the underlying condition, and acute nutritional deficiency from poor oral intake over the preceding week likely triggered the onset of WE in a patient with depleted thiamine stores. This case underscores the importance of considering WE in elderly patients with risk factors such as gastrointestinal surgery, even in the absence of alcohol consumption.

## Introduction

Wernicke encephalopathy (WE) is a serious neurological disorder caused by thiamine (vitamin B1) deficiency and has traditionally been associated with chronic alcohol use. However, an increasing number of non-alcoholic Wernicke encephalopathy (NAWE) cases have been reported in diverse clinical contexts, including malignancies, hyperemesis gravidarum, prolonged fasting, gastrointestinal surgeries, and malnutrition [1].

Although a systematic review has identified gastrointestinal surgical procedures as significant risk factors for NAWE [2], the quantitative risk remains uncharacterized, and detailed descriptions of the clinical course leading to symptom onset are lacking.

In clinical practice, the classical triad of WE - ophthalmoplegia or nystagmus, cerebellar ataxia, and altered mental status - is rarely observed in its entirety. This rarity poses a particular diagnostic challenge in patients without a history of alcohol use [3]. Moreover, serum thiamine levels do not reliably reflect cerebral thiamine status [4], and magnetic resonance imaging (MRI) findings may be negative even in clinically evident cases [5].

Given these diagnostic limitations, NAWE remains underrecognized, especially in patients lacking the complete triad. Here, we report a rare case of NAWE in an elderly woman who had undergone total gastrectomy and subsequently developed symptoms during postoperative rehabilitation in a medical facility. This case underscores the importance of heightened clinical suspicion and offers valuable insight into the diagnostic process for NAWE in the non-alcoholic population.

## Case presentation

An 82-year-old woman presented with decreased activities of daily living (ADL), altered mental status, and a recent history of minor head trauma due to a fall. Her past medical history included hypertension and a previous lacunar infarction without residual deficits. Of particular note, she had undergone a total gastrectomy 22 years earlier for advanced gastric cancer. Her regular medications included aspirin (100 mg), esomeprazole (20 mg), and valsartan (80 mg). Before the onset of symptoms, she had been fully independent in daily life, with no history of dietary restrictions or alcohol consumption.

Approximately three months before presentation, she experienced a fall at home and was diagnosed with a femoral neck fracture. She underwent surgical repair and was subsequently admitted to another hospital for postoperative rehabilitation. One week before transfer to our hospital, she fell again at the rehabilitation facility. Following this second fall, she exhibited poor oral intake, progressive weakness, and ultimately developed altered consciousness. She was therefore transferred to our emergency department for further evaluation and management.

Ethics statement

This case report was prepared for submission to an open-access journal with both institutional opt-out procedures and written informed consent obtained directly from the patient. Patient information has been thoroughly anonymized.

Physical examination

On admission, the patient measured 147 cm in height and weighed 30.4 kg, with a body mass index (BMI) of 14.0, indicating severe undernutrition. Her body temperature was 36.9 °C, blood pressure was 95/56 mmHg, and pulse rate was 103 beats per minute. Both pupils were 3 mm in diameter and reactive to light. Neurologically, she was in a state of impaired consciousness with a Glasgow Coma Scale score of E3V4M6. She was non-ambulatory. The finger-to-nose test revealed marked dysmetria, suggesting cerebellar ataxia. Horizontal nystagmus to the right was also observed.

Investigations

Initial laboratory tests revealed elevated blood urea nitrogen (BUN) at 47.7 mg/dL, suggesting dehydration. There were no findings indicative of hypoglycemia, hyperglycemia, endocrine disorders, electrolyte imbalances, or infections that could account for the altered mental status. Hypoalbuminemia (3.1 g/dL) was also present, suggesting malnutrition. A low serum thiamine level of 1.2 μg/dL was confirmed several days later (Table 1).

**Table 1:** Laboratory tests on admission. WBC, white blood cell count; Hgb, hemoglobin; Plt, platelet count; CRP, C-reactive protein; TP, total protein; Alb, albumin; AST, aspartate aminotransferase; ALT, alanine aminotransferase; LDH, lactate dehydrogenase; CK, creatine kinase; T-Bil, total bilirubin; BUN, blood urea nitrogen; Cre, creatinine; Na, sodium; K, potassium; Cl, chloride; eGFR, estimated glomerular filtration rate; Ca, calcium; P, phosphate; Mg, magnesium; Glu, glucose; PT, prothrombin time; APTT, activated partial thromboplastin time; TSH, thyroid-stimulating hormone; IU, international unit

Parameter	Value (Unit)	Reference range (Unit)
WBC	9.79 × 10^3^/μL	3.3-8.6× 10^3^/μL
Hgb	11.6 g/dL	11.6-14.8 g/dL
Plt	226 × 10^3^/μL	158-348× 10^3^/μL
CRP	0.686 mg/dL	0-0.14 mg/dL
TP	6.6 g/dL	6.6-8.1 g/dL
Alb	3.1 g/dL	4.1-5.1 g/dL
AST	25 IU/L	13-30 IU/L
ALT	27 IU/L	7-23 IU/L
LDH	272 IU/L	124-222 IU/L
CK	83 IU/L	41-153 IU/L
T-Bil	0.57 mg/dL	0.4-1.5 mg/dL
BUN	47.7 mg/dL	8-20 mg/dL
Cre	1.06 mg/dL	0.46-0.79 mg/dL
Na	144 mEq/L	138-145 mEq/L
K	3.5 mEq/L	3.6-4.8 mEq/L
Cl	105 mEq/L	101-108 mEq/L
Ca	9.1 mg/dL	8.8-10.1 mg/dL
P	3.8 mg/dL	2.7-4.6 mg/dL
Mg	2.1 mg/dL	1.8-2.4 mg/dL
Glu	226 mg/dL	73-109 mg/dL
PT activity	85.5%	70-130%
APTT	23.3 sec	24-34 sec
TSH	0.67 μIU/mL	0.61-4.23 μIU/mL
Free thyroxine	0.90 ng/dL	0.77-1.59 ng/dL
Ammonia	63 μg/dL	12-66 μg/dL
Thiamine	1.2 μg/dL	2.6-5.8 μg/dL

**Figure 1:**
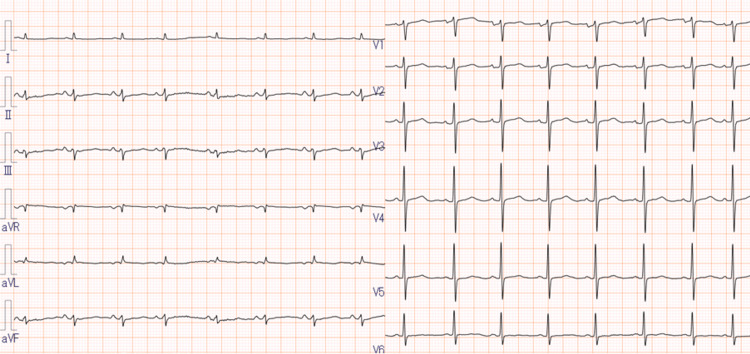
Electrocardiography on admission. Normal sinus rhythm, with no features suggestive of beriberi.

**Figure 2:**
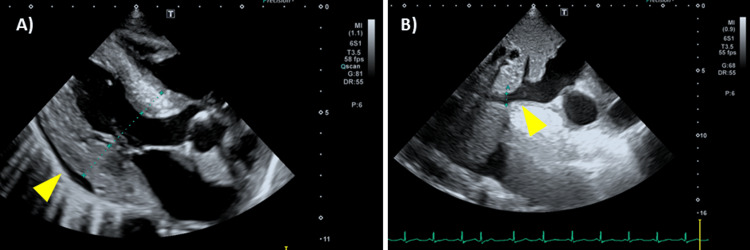
Transthoracic echocardiography on admission. (A) Three-chamber view showing a small pericardial effusion (arrow) and concentric left ventricular wall thickening (IVSd 13.5 mm, PWd 17.5 mm). Left ventricular wall motion is preserved, with an estimated ejection fraction of 60%. No significant valvular disease is observed (mild MR, AR, and TR regurgitation). (B) The inferior vena cava (IVC) diameter is 9 mm, with good respiratory variation (arrow). MR, mitral regurgitation; AR, aortic regurgitation; TR, tricuspid regurgitation; IVSd, interventricular septum diameter; PWd, posterior wall diameter

**Figure 3:**
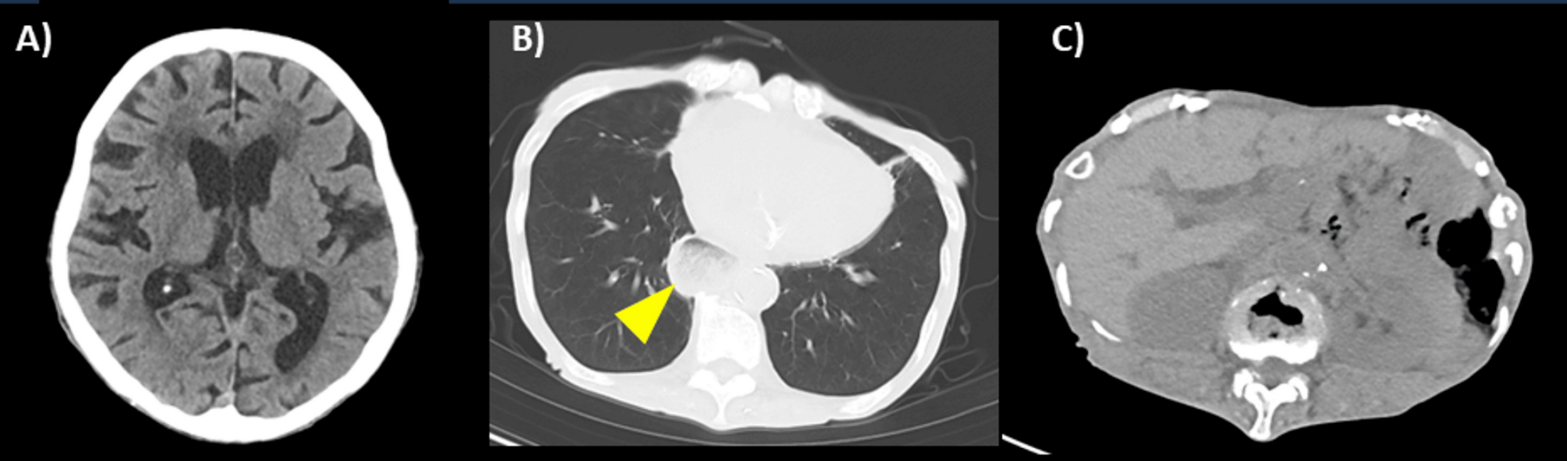
Non-contrast computed tomography on admission. (A) No evidence of intracranial hemorrhage or space-occupying lesion. (B) No signs of trauma or pneumonia; a hiatal hernia is present (arrow). (C) Minimal visceral fat and poor delineation of intra-abdominal organs, consistent with post-gastrectomy status. No ascites or traumatic findings are observed.

**Figure 4:**
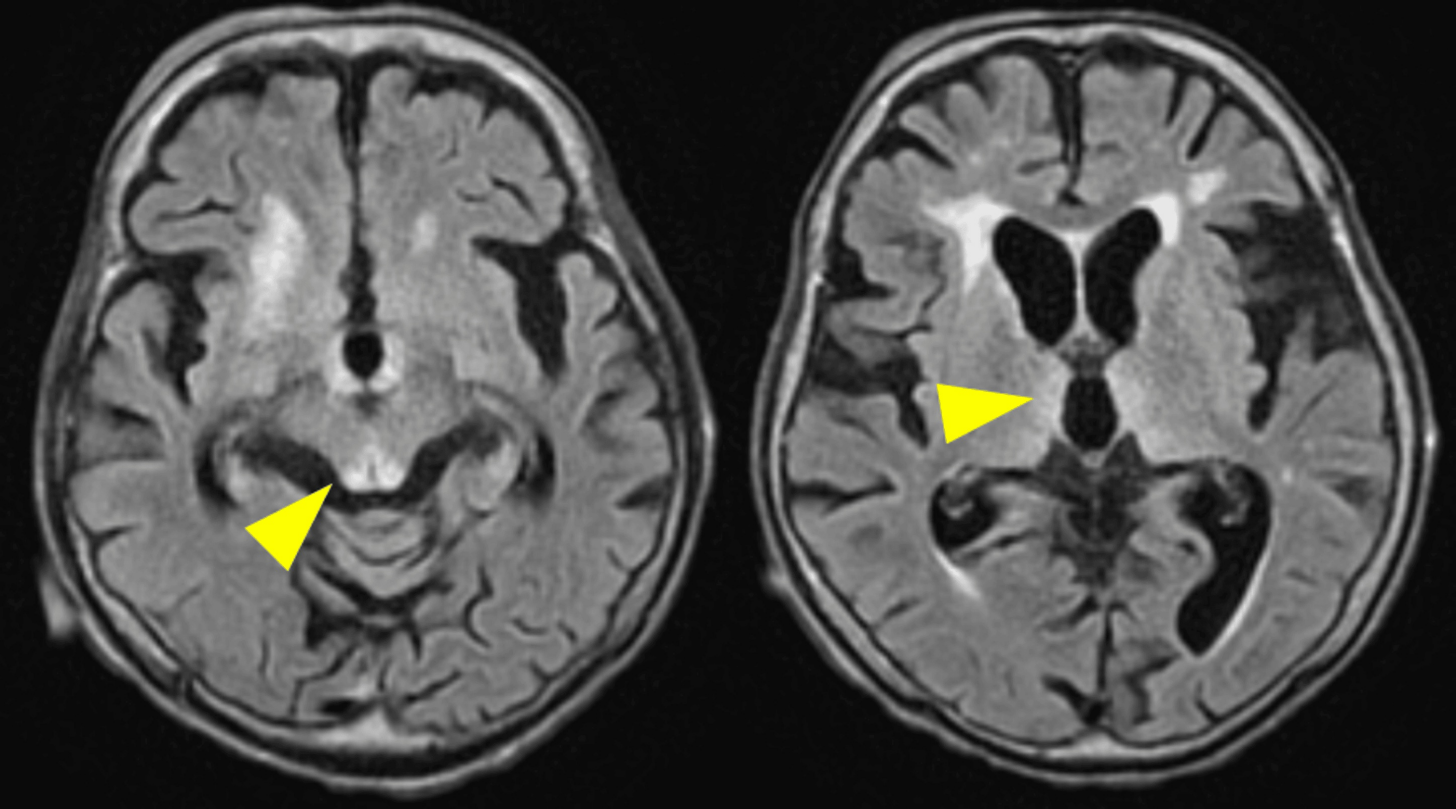
Brain magnetic resonance imaging on admission. Fluid-attenuated inversion recovery (FLAIR) images demonstrate symmetrical high-intensity lesions in the periaqueductal gray matter and bilateral medial thalami, consistent with Wernicke encephalopathy (arrows).

Electrocardiography showed a normal sinus rhythm without features suggestive of wet beriberi (Figure 1). Transthoracic echocardiography revealed collapse of the left ventricle and inferior vena cava, consistent with intravascular volume depletion. Although a small pericardial effusion was observed, left ventricular wall motion and cardiac output were preserved (Figure 2). Non-contrast computed tomography (CT) of the head and trunk showed no evidence of trauma-related lesions or findings consistent with encephalopathy (Figure 3). Brain MRI revealed symmetric high-intensity signals in the periaqueductal gray matter and bilateral medial thalami on fluid-attenuated inversion recovery (FLAIR) sequences (Figure 4), findings characteristic of WE.

Clinical course

Following two episodes of falling, the patient developed poor oral intake, progressive muscle weakness, and altered consciousness. Based on this clinical course, intracranial pathologies such as subdural hematoma and metabolic disturbances, including hyponatremia or glucose abnormalities, were initially suspected. However, these conditions were ruled out through cranial CT and laboratory tests conducted in the emergency department. Subsequent physical examination revealed ophthalmoplegia or nystagmus, cerebellar ataxia, and altered mental status, prompting a brain MRI, which confirmed the diagnosis of WE. The patient’s low BMI (14.0 kg/m^2^), hypoalbuminemia, and prior total gastrectomy were considered contributory findings in support of the diagnosis. Although the presence of pericardial effusion raised the possibility of beriberi heart disease, the timing of its onset was unclear, and the volume was minimal. Electrocardiography did not reveal any significant abnormalities, and a conservative approach with close monitoring was adopted.

Following admission, the patient’s level of consciousness slightly improved with intravenous fluid therapy for dehydration. Given the history, physical findings, laboratory data, and MRI features, a clinical diagnosis of WE was made, and thiamine supplementation was initiated without waiting for serum thiamine results.

**Figure 5:**
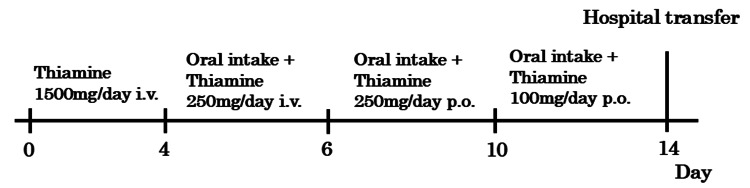
Clinical course. Days 0-4: Thiamine 1500 mg/day i.v.; days 4-6: consciousness improved; oral intake resumed + Thiamine 250 mg/day i.v.; days 6-10: switched to oral Thiamine 250 mg/day p.o.; days 10-14: reduced to Thiamine 100 mg/day p.o.; and day 14: transferred for rehabilitation i.v., intravenous injection; p.o., per os, i.e., oral medication

High-dose intravenous thiamine therapy was started on day 1 at 1,500 mg/day for three days. With continued fluid replacement and thiamine administration, the patient’s mental status gradually improved. On day 4, the dosage was reduced to 250 mg/day, and oral intake was resumed. By day 6, thiamine was transitioned to oral administration at 250 mg/day. Continued clinical improvement was observed, and the dose was further tapered to 100 mg/day on day 10. On day 14, thiamine supplementation was completed, and the patient was transferred to a rehabilitation facility in a stable condition (Figure 5).

## Discussion

WE is a neurological emergency caused by thiamine (vitamin B1) deficiency. Thiamine functions as an essential coenzyme in carbohydrate metabolism, acting as a cofactor for key enzymes in the citric acid cycle and the pentose phosphate pathway. Its deficiency leads to impaired oxidative metabolism and energy failure, particularly in vulnerable brain regions such as the periaqueductal gray matter and medial thalamus [4]. On MRI, characteristic findings include FLAIR hyperintensities in these regions [6]. Although one study reported a 100% positivity rate for MRI findings in NAWE cases (17 out of 17 patients) [7], the overall sensitivity of MRI in detecting WE, including atypical presentations, is generally estimated at approximately 70%-80% [5]. Therefore, it is critical to recognize that a negative MRI does not rule out the diagnosis of WE in clinical practice. Notably, serum thiamine levels do not reliably reflect cerebral thiamine status, and normal levels cannot definitively exclude the diagnosis of WE [4]. Moreover, the availability of rapid serum thiamine assays is limited in many clinical settings. Therefore, clinicians should avoid relying solely on thiamine levels to initiate diagnosis or treatment, as this may lead to delays in appropriate management.

Traditionally, WE has been strongly associated with chronic alcohol use, but non-alcoholic cases are increasingly recognized. Chamorro et al. reported that 92.7% of WE cases were alcohol-related, while non-alcoholic risk factors included gastrointestinal surgery (17.6%), malignancy (8.8%), hyperemesis gravidarum (8.8%), and prolonged fasting (5.9%) [1].

Gastrointestinal surgical procedures, including total gastrectomy, are established risk factors for thiamine deficiency and have been identified as contributors to NAWE [2]. However, to date, no systematic reviews have specifically evaluated WE cases following total gastrectomy, and risk stratification based on specific surgical procedures remains lacking. Mechanistically, thiamine absorption may be impaired due to loss of gastric acid, alterations in gut microbiota, or overgrowth of thiaminase-producing bacteria [8]. Although the jejunum remains the primary site of thiamine absorption, inadequate gastric processing and food intake may result in chronic depletion. The body's total thiamine stores are approximately 25-30 mg, with a daily requirement of 1.1-1.4 mg in healthy adults. Thus, deficiency can occur within two to three weeks of insufficient intake [9].

In the present case, chronic thiamine depletion due to prior total gastrectomy, combined with acute dietary insufficiency following repeated falls during rehabilitation, likely triggered the onset of WE. This highlights that even a short period of poor oral intake can precipitate WE in predisposed individuals. The patient's markedly low BMI (14 kg/m²) supported the underlying malnourished state, and notably, the classic triad of WE - ophthalmoplegia or nystagmus, cerebellar ataxia, and altered mental status - was fully present. These features are rarely observed together, occurring in only 16%-17% of cases [3]. Harper et al. also reported that only 20% of pathologically confirmed cases had been clinically diagnosed before death, underscoring the difficulty of timely recognition [10].

This case also raises awareness that WE can occur in atypical settings. The patient developed symptoms during inpatient rehabilitation after orthopedic surgery, with only a short period of reduced intake, suggesting that current clinical assumptions about patient risk profiles may need to be reconsidered. The recognition of WE in such settings is critical for prompt treatment and prevention of permanent neurological sequelae.

Several limitations should be acknowledged. Evaluation of other micronutrient and vitamin deficiencies was not performed; thus, the contribution of factors other than thiamine cannot be ruled out. Additionally, long-term neurological follow-up data were unavailable at the time of reporting, limiting assessment of the full clinical trajectory.

## Conclusions

We report a rare case of NAWE in an elderly woman with a history of total gastrectomy who developed acute neurological symptoms during postoperative rehabilitation for a femoral neck fracture. This case highlights the importance of early recognition of thiamine deficiency in malnourished patients with a history of gastrointestinal surgery, even in the absence of alcohol use and even among hospitalized patients.
